# Validation of the ALBI-TAE model and comparison of seven scoring systems for predicting survival outcome in patients with intermediate‐stage hepatocellular carcinoma undergoing chemoembolization

**DOI:** 10.1186/s40644-023-00575-6

**Published:** 2023-05-30

**Authors:** Kittipitch Bannangkoon, Keerati Hongsakul, Teeravut Tubtawee

**Affiliations:** Department of Radiology, Faculty of Medicine, Prince of Songkla University, Hat Yai, Songkhla, 90110 Thailand

**Keywords:** Hepatocellular carcinoma, Transarterial chemoembolization, Model, Prediction, Prognosis, Survival rate

## Abstract

**Background:**

The ALBI-TAE model was recently proposed as a scoring system to select suitable patients with intermediate-stage hepatocellular carcinoma (HCC) for transarterial chemoembolization (TACE). However, this scoring system has not been externally validated. Therefore, we validated this score and compared it with six scoring systems in terms of prognostication.

**Methods:**

We retrospectively enrolled 480 patients with intermediate-stage HCC who underwent TACE at a tertiary care center between January 2008 and December 2019. Seven scores, which included the ALBI-TAE model, Bolondi’s subclassification, HAP score, mHAP-II score, tumor burden score, six-and-twelve score, and seven-eleven criteria, were calculated and a head-to-head comparison was made in terms of prognostic power using Harrell’s C-index. Prognostic factors associated with survival were analyzed.

**Results:**

ALBI-TAE group A had the longest median overall survival (OS) of 40.80 months, followed by ALBI-TAE groups B, C, and D of 20.14 months, 10.58 months, and 7.54 months, respectively, with significant differences (*P* < 0.001). Among the seven scores, the ALBI-TAE model had the best predictive performance (Harrell’s C-index 0.633) in differentiating OS in intermediate-stage HCC patients. Moreover, the ALBI-TAE model was identified as an independent prognostic factor for survival outcome in multivariate analysis.

**Conclusion:**

Our study confirmed the value of the ALBI-TAE model with excellent prognostic discriminatory power in intermediate-stage HCC patients. The ALBI-TAE model is a simple and valuable predictive tool to identify patients with good prognosis who can get the most benefit from TACE.

## Introduction

Hepatocellular carcinoma (HCC) is the third leading cause of cancer death worldwide with approximately 830,000 deaths in 2020 [1]. Even though advancements have been made in screening systems and treatment modalities, the prognosis of HCC patients is poor because a high proportion of patients had large and multiple tumors at the initial presentation [2]. The treatment strategies of HCC are stratified according to Barcelona Clinic Liver Cancer (BCLC) stages [3, 4]. Intermediate-stage HCC (BCLC-B) is comprised of patients with multinodular tumor burden, preserved liver function, and good performance status [3, 4]. Transarterial chemoembolization (TACE), which consists of injecting chemotherapeutic agents emulsified with lipiodol into the tumor feeding arteries followed by embolic agents, is the primary recommended therapy in BCLC-B based on two randomized trials [5, 6].

The recent update of the BCLC staging system in 2022 restricts the recommendation for TACE in patients who have well-defined tumor nodules, preserved portal flow, and selective vascular access and are not eligible for liver transplantation [7]. On the other hand, patients with diffuse, infiltrative, and extensive bilobar involvement should be better candidates for systemic therapy as the first-line treatment [7]. Categorization of patients in this subgroup of BCLC-B remains unclear and no cutoff point has been published. Additionally, because of the very heterogeneous nature of this population with a wide range of tumor burdens, it is necessary to subclassify BCLC-B to facilitate more appropriate treatment strategies in clinical practice.

Conventionally, the selection of treatment for HCC relies closely on the size and number of tumor nodules. The six-and-twelve (SAT) score and seven-eleven criteria (SEC) were proposed to assess tumor burden in HCC patients [8, 9]. These two prognostic scores are calculated by combining the largest diameter of the nodules and the number of tumors. The three strata of these scores include the summation of scores of ≤ 6, > 6 up to 12, and > 12 in the SAT score and ≤ 7, > 7 up to 11, and > 11 in the SEC. The higher strata predict lower patient survival outcome. More recently, the tumor burden score in HCC patients who undergo TACE was reported [10, 11]. In contrast to the SAT score and SEC, the tumor burden score integrates both tumor number and tumor size into a single continuous variable and was shown to differentiate the prognosis among HCC patients.

Furthermore, some of the predictive scores need a sophisticated calculation of tumor burden and liver function. The proposed Bolondi’s subclassification system for intermediate HCC, based on the Child–Pugh score, tumor burden, performance status, and presence of portal vein thrombosis, has been applied to Asian and European cohorts [12–15]. Recently, the hepatoma arterial-embolization prognostic (HAP) score and the modified HAP-II (mHAP-II) score that contain four variables (serum alpha-fetoprotein [AFP], tumor burden, serum albumin, and total serum bilirubin) have also been proposed with significant prognostic performance in selection of optimal candidates for TACE [16, 17].

Recently, the ALBI-TAE model was introduced for accurate prognostication and selection of HCC patients who can benefit the most from TACE [18]. This prognostic model was developed at a large-volume medical center in Taiwan. The study reported that the combination of three factors, which included the up-to-11 criteria, serum AFP, and albumin-bilirubin (ALBI) grade, was an independent predictor to estimate overall survival (OS) in BCLC-B HCC patients undergoing TACE. The four strata of the model include class A (low risk), B (intermediate risk), C (high risk), and D (very high risk). An internal validation was achieved in Taiwanese patients.

To the best of our knowledge, no study has published an external validation of the recent ALBI-TAE model in HCC patients undergoing TACE. Thus, we aimed to validate the ALBI-TAE model in Thai HCC patients with BCLC-B stage and compare this score with several established scoring systems in terms of prognostic power.

## Methods 

### Patient population

This study was carried out in compliance with the ethical principles outlined in the Declaration of Helsinki and was approved by the institutional ethics committee (REC No. 65–269-7–1). The requirement for written informed consent for this study was waived by the Institutional Review Board, and all data were analyzed anonymously. Patients with unresectable HCC who underwent conventional TACE as the first-line treatment between January 2008 and December 2019 were included in this study. HCC was diagnosed based on histopathological results by liver biopsy or radiological characteristics by the assessment of dynamic contrast-enhanced computed tomography (CT) and magnetic resonance imaging (MRI) according to the diagnostic criteria of the American Association for the Study of Liver Diseases guidelines [3]. The inclusion criteria for our study were as follows: (i) age > 18 years; (ii) intermediate-stage HCC (BCLC-B) defined as tumor size > 3 cm, tumor number > 3, or a single tumor > 5 cm without vascular invasion or extrahepatic metastasis; and (iii) Child–Pugh class A and B. The exclusion criteria were (i) initial treatment with tumor resection; (ii) locoregional or systemic therapies before the TACE session; (iii) diffuse infiltrative tumor; (iv) concomitant malignancy; (v) history of spontaneous tumor rupture; and (vi) no evaluation using contrast-enhanced imaging.

### Transarterial chemoembolization protocols

All eligible HCC patients underwent TACE using digital subtraction angiography (DSA) guidance (Allura Clarity FD20, Philips Healthcare, Eindhoven, the Netherlands) under the supervision of experienced interventional radiologists through the transfemoral route. A microcatheter (1.7-Fr to 2.4-Fr) was advanced through a 5-Fr catheter inserted into the celiac artery and contrast medium was injected into the common hepatic artery to identify the tumor-feeding arteries. We performed selective catheterization as distal as possible into the tumor-feeding branches. In the next step, we slowly administered a mixture of iodized oil (range 2‒16 mL) (Lipiodol, Guerbet) and doxorubicin hydrochloride (range 5–50 mg) (Adriamycin, Pfizer) or mitomycin (range 10‒20 mg) (Vesimycin, Naprod Life Sciences) followed by embolization of the branches using gelatin sponge particles. We finished the procedure when the tumor-feeding branch was entirely blocked and disappearance of tumor staining from DSA was observed.

### Treatment evaluation and follow-up

An imaging study using 4-phase contrast-enhanced CT scan or dynamic MRI was carried out within 1 month after the initial procedure to evaluate the radiological response of the treated tumors according to the modified Response Evaluation Criteria in Solid Tumors (mRECIST) [19]. If no definite evidence of residual or recurrent tumor was seen, imaging investigation was evaluated subsequently at 3-month intervals. The decision to repeat the TACE procedure was carried out on demand based on patterns of tumor recurrence, BCLC staging, and preserved hepatic reserve.

### Data collection

The following data were collected: demographic data that included age and sex; clinical history (alcohol consumption, hepatitis B or C virus carriers, diabetes mellitus, presence of ascites, and Child–Pugh class); laboratory data (serum AFP, levels of alanine transaminase, albumin, total bilirubin, and platelet count); tumor factors (size and number of tumors); imaging response within one month after the initial TACE; and complications (post embolization syndrome and liver decompensation). Postembolization syndrome was defined as a set of symptoms that includes nausea, vomiting, abdominal pain, and fever over 38 °C occurring after TACE. The development of liver decompensation was defined as the occurrence of any of the following within 4 weeks after TACE: an increase in serum bilirubin levels of ≥ 2.0 mg/dL or an increase of at least 3 times the baseline or upper limit of normal, the development of new or increasing ascites or encephalopathy. OS was calculated from the date of initial TACE received until death from any cause or the censoring date of December 31, 2021. Survival data were obtained from national statistical data provided by the Thailand civil registration database. The ALBI-TAE model, Bolondi’s subclassification for BCLC-B, HAP score, mHAP-II score, tumor burden score, SAT score, and SEC were calculated as described in the original publications [8–10, 12, 16–18] (Table 1).

**Table 1:** Calculations of the seven scoring models to predict TACE response for intermediate-stage HCC patients

Score	Parameters included		Points	Risk
ALBI-TAE score	ALBI grade	2–3	0	A (Low risk)
	AFP	> 200 ng/mL	1	B
	Up-to-11	Out	2	C
			3	D (Very high risk)
Bolondi’s subclassification for BCLC-B	Child–Pugh score (CPS)	CPS 5–7, Up-to-7: within, ECOG 0		B1 (Low risk)
	Up-to-7	CPS 5–6, Up-to-7: beyond, ECOG 0		B2
	ECOG status	CPS 7, Up-to-7: beyond, ECOG 0		B3
	No portal vein thrombosis	CPS 8–9, Up-to-7: any, ECOG 0–1		B4 (High risk)
HAP score	Tumor size	> 7 cm	0	A (Low risk)
	AFP	> 400 ng/mL	1	B
	Albumin	< 36 g/L	2	C
	Bilirubin	> 17 µmol/L	≥ 3	D (High risk)
mHAP-II score	Tumor size	> 7 cm	0	A (Low risk)
	Tumor number	≥ 2 lesions	1	B
	AFP	> 400 ng/mL	2	C
	Albumin	< 36 g/L	≥ 3	D (High risk)
	Bilirubin	> 17 µmol/L		
Tumor burden score	Largest tumor diameter (cm)	Square root [(largest tumor diameter)^2^ + (number of tumors)^2^]	< 3.36	A (Low risk)
	Number of tumors		3.36–13.74	B
			> 13.74	C (High risk)
Six-and-twelve score	Largest tumor diameter (cm)	Sum	≤ 6	A (Low risk)
	Tumor numbers		7–12	B
			> 12	C (High risk)
Seven-eleven criteria	Largest tumor diameter (cm)	Sum	≤ 7	A (Low risk)
	Tumor numbers		7–11	B
			> 11	C (High risk)

*Abbreviations*: *TACE* Transarterial chemoembolization, *HCC* Hepatocellular carcinoma, *ALBI* Albumin-bilirubin grade, *AFP* Alpha-fetoprotein, *BCLC* Barcelona Clinic Liver Cancer, *ECOG*, Eastern Cooperative Oncology Group, *HAP* Hepatoma arterial-embolization prognostic, *mHAP-II* Modified HAP-II

### Statistical analysis

Baseline characteristics are presented as median with interquartile range (IQR) for skewed distribution or mean ± standard deviation (SD) for normally distributed variables. Categorical variables are presented as frequency with percentages. Prognostic factors affecting survival were evaluated by univariate analysis. Subsequently, all factors with *P* < 0.20 in the univariate analysis were included in the multivariate analysis. Multivariate Cox regression analysis with backward selection was made to detect independent predictors of survival time. Entry criteria for selection into the final multivariate model was *P* < 0.05. Survival curves were estimated for each group of ALBI-TAE model using the Kaplan–Meier method and compared statistically using the log rank test. The ALBI-TAE model was validated and compared with the other scores, including Bolondi’s subclassification for BCLC-B [12], HAP score [16], mHAP-II score [17], tumor burden score [10], SAT score [8], and the SEC [9]. Head-to-head comparisons between the prognostic scores were assessed using Harrell’s C-index and prediction error curves based on the integrated Brier score (IBS). A C-Index of 0.5 indicates no predictive power and a C-Index of 1.0 implies perfect predictive ability. The prediction error was made by calculating the IBS over the study interval of 0‒60 months. All statistical analyses were achieved with R software, version 4.2.0 (R Foundation, Vienna, Austria).

## Results 

### Baseline characteristics

The study included 480 patients who met the full set of inclusion and exclusion criteria. Their baseline patient characteristics are displayed in Table 2. The mean age was 62 years, and 72% were men. The main etiology of HCC was hepatitis B virus (54%) followed by hepatitis C virus (21%), and alcohol (17%). The Child–Pugh classes were A (66%) and B (34%). At the initial diagnosis of HCC, the most common largest tumor size was > 5 cm (60%) followed by 3–5 cm (26%). Tumor sizes ranged 1.0–21.7 cm and the mean tumor size ± SD was 7.2 ± 4.6 cm. More than half (55%) of the patients had 2–5 tumor nodules. Median values for serum alanine transaminase and platelet count were 40 (27–61) U/L and 122 × 10^3^/mm^3^ (76–201), respectively. Most patients had serum AFP ≤ 200 ng/mL (63%). The median (IQR) serum albumin and total bilirubin levels were 3.4 (3.0,3.8) g/dL and 0.83 (0.54,1.39) mg/dL, respectively. Tumor response rates by mRECIST with complete response, partial response, stable disease, and progressive disease were 15% (74 patients), 41% (195 patients), 25% (118 patients), and 19% (93 patients), respectively. In addition, most patients had ALBI-TAE grade B (45%).

**Table 2:** Baseline patient demographic and clinical characteristics of 480 patients with intermediate-stage HCC who underwent TACE

Variables	All patients
Age (years), mean ± standard deviation	62 ± 11
Male/female, n (%)	358/112 (75/25)
Alcohol consumption, n (%)	80 (17)
Hepatitis B carrier, n (%)	261 (54)
Hepatitis C carrier, n (%)	99 (21)
Diabetes mellitus, n (%)	92 (19)
Size of the largest lesion	
< 3 cm, n (%)	66 (14)
3–5 cm, n (%)	125 (26)
> 5 cm, n (%)	289 (60)
Tumor number	
Single, n (%)	136 (28)
2–5, n (%)	264 (55)
> 5, n (%)	80 (17)
Serum AFP level ≤ 200 ng/mL, n (%)	302 (63)
Ascites, n (%)	48 (10)
Alanine transaminase (U/L), median (IQR)	40 (27–61)
Albumin (g/dl), median (IQR)	3.4 (3.0–3.8)
Total bilirubin (mg/dL), median (IQR)	0.83 (0.54–1.39)
Platelet count (× 10^3^/mm^3^), median (IQR)	122 (76–201)
Child–Pugh class (A/B), n (%)	317/163 (66/34)
ALBI grade, n (%)	
1	123 (26)
2	305 (63)
3	52 (11)
Treatment response to initial TACE	
CR/PR/SD/PD, n (%)	74/195/118/93 (15/41/25/19)
ALBI-TAE model	
A (Low risk)	44 (9)
B (Intermediate risk)	216 (45)
C (High risk)	164 (34)
D (Very high risk)	56 (12)

*Abbreviations*: *AFP* Alpha-fetoprotein, *IQR* Interquartile range, *CR* Complete response, *PR* Partial response, *SD* Stable disease, *PD* Progressive disease

### Analysis of the prognostic factors for overall survival (OS)

In univariate analysis, size of the largest lesion, number of tumors, serum AFP level, serum albumin level, serum total bilirubin level, Child–Pugh class, treatment response to initial TACE, and ALBI-TAE model were associated with poor OS. Multivariate Cox model showed that Child–Pugh class B (hazard ratio [HR] 1.52, 95% CI 1.23–1.87; *P* < 0.001), treatment response to initial TACE (HR 1.65, 95% CI 1.35–2.01; *P* < 0.001), and ALBI-TAE model (group B vs. group A [HR 1.59, 95% CI 1.06–2.40: *P* = 0.026], group C vs. group A [HR 2.54, 95% CI 1.66–3.88; *P* < 0.001], and group D vs. group A [HR 3.73, 95% CI 2.31–6.01; *P* < 0.001]) were independently associated with increased mortality in these patients (Table 3).

**Table 3:** Univariate and multivariate analysis of prognostic factors associated with overall survival

**Prognostic Factors**	**Reference**	**Univariate Analysis**		**Multivariate Analysis**	
		**HR (95% CI)**	***P***	**HR (95% CI)**	***P***
Age, > 60 years	≤ 60 years	0.94 (0.78–1.14)	0.529		
Sex, female	male	1.16 (0.93–1.44)	0.184		
Alcohol consumption, yes	No	0.83 (0.64–1.08)	0.169		
Hepatitis B carrier, positive	Negative	1.09 (0.90–1.32)	0.386		
Hepatitis C carrier, positive	Negative	1.06 (0.84–1.34)	0.634		
Diabetes mellitus, yes	No	1.14 (0.90–1.45)	0.279		
Size of the largest lesion, > 7 cm	≤ 7 cm	1.49 (1.23–1.81)	< 0.001		
Tumor number, > 5	≤ 5	1.60 (1.25–2.05)	< 0.001		
Serum AFP level, > 200 ng/mL	≤ 200 ng/mL	1.56 (1.28–1.90)	< 0.001		
Ascites, present	absent	1.34 (0.98–1.84)	0.071		
Alanine transaminase, > 40 U/l	≤ 40 U/L	1.17 (0.97–1.42)	0.101		
Albumin, < 3.6 g/dL	≥ 3.6 g/dL	1.61 (1.31–1.98)	< 0.001		
Total bilirubin, > 1.2 mg/dL	≤ 1.2 mg/dL	1.41 (1.15–1.73)	< 0.001		
Platelet count, ≤ 10^3^ mm^3^	> 10^3^ mm^3^	1.03 (0.84,1.25)	0.797		
Child–Pugh class B	class A	1.48 (1.22–1.81)	< 0.001	1.52 (1.23–1.87)	< 0.001
Treatment response to TACE, SD + PD	CR + PR	1.91 (1.58–2.32)	< 0.001	1.65 (1.35–2.01)	< 0.001
ALBI-TAE model	A (Low risk)				
B (Intermediate risk)		2.08 (1.40–3.09)	< 0.001	1.59 (1.06–2.40)	0.026
C (High risk)		3.46 (2.30–5.21)	< 0.001	2.54 (1.66–3.88)	< 0.001
D (Very high risk)		5.10 (3.22–8.09)	< 0.001	3.73 (2.31–6.01)	< 0.001

*Abbreviations*: *HR* Hazard ratio, *CI* Confidence level, *AFP* Alpha-fetoprotein, *TACE* Transarterial chemoembolization, *SD* Stable disease, *PD* Progressive disease, *CR* Complete response, *PR* Partial response

### Survival analysis

**Fig. 1:**
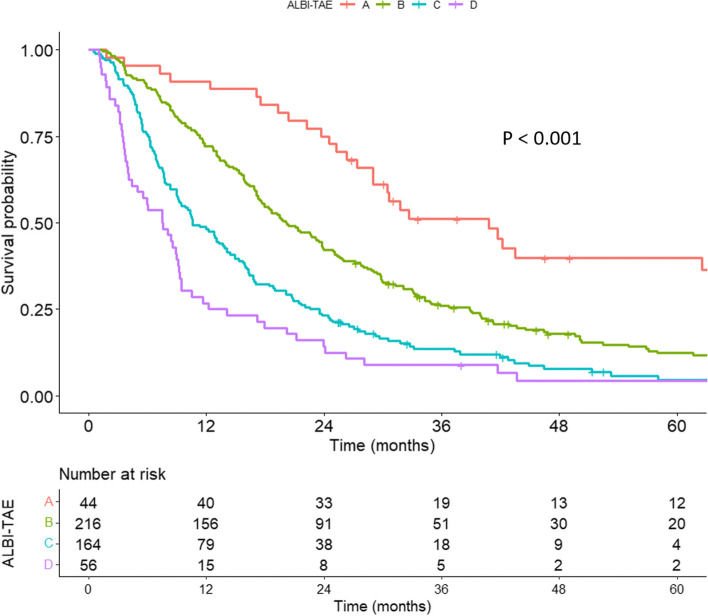
Kaplan–Meier curves of overall survival among intermediate-stage HCC patients who underwent TACE stratified by the ALBI-TAE model

The median (IQR) OS of the entire cohort was 16.6 months (14.9,18.4 months). The 1-, 3-, and 5-year OS rates were 60%, 22%, and 11% respectively. By stratifying the ALBI-TAE model (groups A, B, C, and D), the median (IQR) OS rates were 40.80 (29.04,105.19), 20.14 (17.61,23.85), 10.58 (9.17,14.13), and 7.54 (4.37,9.33) months (Fig. 1). The 1-, 3-, and 5-year OS rates were 91%, 51%, and 40%, respectively, for ALBI-TAE A; 72%, 26%, and 12%, respectively, for ALBI-TAE B; 48%, 14%, and 5%, respectively, for ALBI-TAE C; and 27%, 9%, and 4%, respectively, for ALBI-TAE D. There were significant survival differences between the four groups (*P* < 0.001).

### Comparing the performances of the ALBI-TAE model and other scores .

**Fig. 2:**
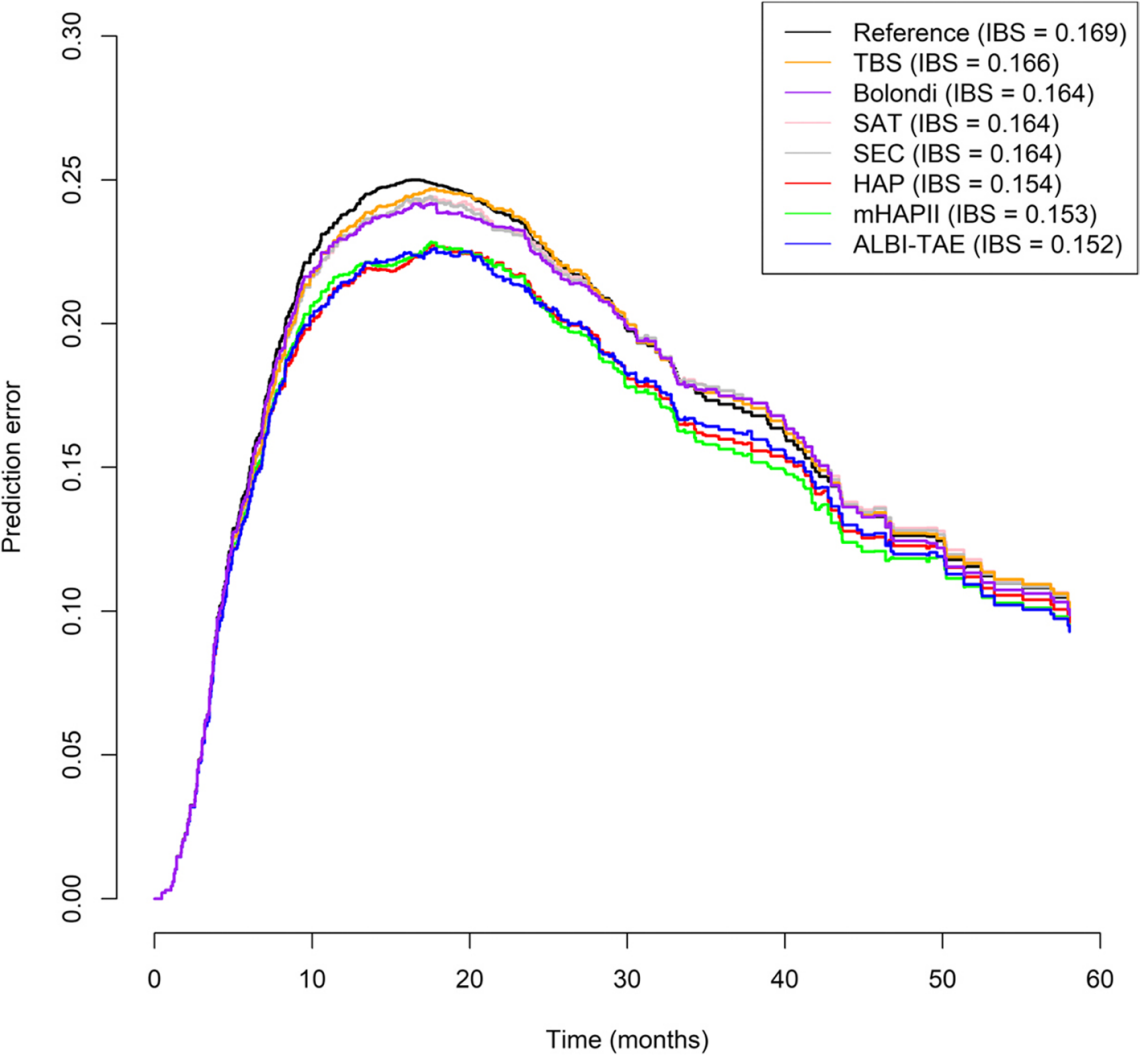
Predictive error curve and integrated Brier score (IBS) for Kaplan–Meier estimates based on the ALBI-TAE model (blue), mHAP-II score (green), HAP score (red), seven-eleven-criteria (gray), six-and-twelve score (pink), Bolondi’s subclassification (purple), tumor burden score (orange), and compared with the unstratified sample (black)

Table 4 provides a detailed overview of the head-to-head comparison. Among these seven scores, the ALBI-TAE model had the highest C-index of 0.633, which suggested a better prognostic performance to discriminate survival in TACE patients, followed by the HAP score (0.629), mHAP-II score (0.624), SEC (0.578), SAT score (0.574), Bolondi’s subclassification (0.570), and tumor burden score (0.546). The IBS values for the study interval (0–60 months) were 0.152 for the ALBI-TAE model, 0.153 for the mHAP-II score, and 0.154 for the HAP score. Based on the Kaplan Meier estimates of the unstratified sample, the reference IBS was 0.169. The prediction error curves based on the IBS are shown in Fig. 2.

**Table 4:** Head-to-head comparison of the performance and discriminative ability of the seven models in predicting survival for intermediate-stage HCC patients who underwent TACE

Scoring system	Risk	Number	Median (IQR) OS (months)	Hazard Ratio (95% CI)	*P*	Harrell’s C-index
ALBI-TAE score	A (Low risk)	44	40.80 (29.04,105.19)	1	ref	0.633
	B	216	20.14 (17.61,23.85)	2.08 (1.40–3.09)	< 0.001	
	C	164	10.58 (9.17,14.13)	3.46 (2.30–5.21)	< 0.001	
	D (Very high risk)	56	7.54 (4.37,9.33)	5.10 (3.22–8.09)	< 0.001	
Bolondi’s subclassification for BCLC-B	B1 (Low risk)	126	21.83 (17.28,27.1)	1	ref	0.570
	B2	243	17.31 (15.34,20.4)	1.21 (0.96–1.53)	0.102	
	B3	55	9.43 (7.65,12.7)	2.03 (1.45–2.84)	< 0.001	
	B4 (High risk)	56	11.61 (9.07,15.9)	1.66 (1.19–2.32)	0.003	
HAP score	A (Low risk)	62	33.28 (29.01,44.58)	1	ref	0.629
	B	200	18.97 (16.92,23.26)	1.86 (1.34–2.58)	< 0.001	
	C	162	11.66 (8.94,13.83)	2.88 (2.06–4.03)	< 0.001	
	D (High risk)	56	8.23 (7.42,9.46)	3.98 (2.67–5.94)	< 0.001	
mHAP-II score	A (Low risk)	16	49.64 (30.62,NA)	1	ref	0.624
	B	107	27.33 (23.26,33.3)	2.00 (1.04–3.86)	0.039	
	C	181	16.85 (15.70,19.5)	3.15 (1.66–5.98)	< 0.001	
	D (High risk)	176	9.18 (7.75,11.8)	4.80 (2.52–9.12)	< 0.001	
Tumor burden score	A (Low risk)	24	16.92 (9.17,56.9)	1	ref	0.546
	B	378	17.81 (16.26,20.7)	1.19 (0.75–1.86)	0.461	
	C (High risk)	78	8.15 (6.18,13.0)	1.87 (1.14–3.07)	0.014	
Six-and-twelve score	A (Low risk)	77	22.1 (15.77,29.0)	1	ref	0.574
	B	251	19.5 (17.12,23.8)	1.15 (0.87–1.51)	0.335	
	C (High risk)	152	9.4 (8.34,13.0)	1.75 (1.30–2.35)	< 0.001	
Seven-eleven criteria	A (Low risk)	153	21.6 (17.12,25.8)	1	ref	0.578
	B	157	18.3 (15.83,24.0)	1.13 (0.89–1.44)	0.312	
	C (High risk)	170	10.4 (8.94,13.7)	1.65 (1.31–2.08)	< 0.001	
Total		480	16.6 (14.9,18.4)			

*Abbreviations*: *IQR* Interquartile range, *BCLC* Barcelona Clinic Liver Cancer, *HAP* Hepatoma arterial-embolization prognostic, *mHAP-II* Modified HAP-II

### Treatment response and complications stratified by the ALBI-TAE model

Treatment response and complications after TACE were evaluated based on the ALBI-TAE model (Table 5). Among the 480 patients, 269 responded well to TACE, while 211 had a poor response. Compared to ALBI-TAE group A, the chance of TACE response was significantly lower in ALBI-TAE group C (adjusted OR 0.14, 95% CI 0.06–0.34) and ALBI-TAE group D (adjusted OR 0.04, 95% CI 0.01–0.12). Patients in ALBI-TAE group B had a numerically lower chance of TACE response compared to patients in ALBI-TAE group A, although not statistically significant (adjusted OR 0.44, 95% CI 0.19–1.04). Postembolization syndrome was more common in ALBI-TAE groups B (19%), C (31%), and D (59%) compared to patients in ALBI-TAE group A (11%) with significant differences (P < 0.001). Moreover, ALBI-TAE group A had no incidence of liver decompensation, while groups B, C, and D had rates of 5%, 9%, and 29%, respectively. These differences between the groups were significant (P < 0.001).

**Table 5:** Treatment response and complications of intermediate-stage HCC patients who underwent TACE, stratified by the ALBI-TAE model

Post-TACE evaluations	Group A	Group B	Group C	Group D	*P* value
	(*n* = 44)	(*n* = 216)	(*n* = 164)	(*n* = 56)	
mRECIST					< 0.001
CR	11 (25)	48 (22)	14 (9)	1 (2)	
PR	26 (59)	103 (48)	57 (35)	9 (16)	
SD	6 (14)	40 (18)	50 (30)	22 (39)	
PD	1 (2)	25 (12)	43 (26)	24 (43)	
Treatment response					< 0.001
Good response (CR + PR)	37 (84)	151 (70)	71 (43)	10 (18)	
Poor response (SD + PD)	7 (16)	65 (30)	93 (57)	46 (82)	
Complications					
Post embolization syndrome	5 (11)	40 (18)	51 (31)	33 (59)	< 0.001
Liver decompensation	0 (0)	11 (5)	15 (9)	16 (29)	< 0.001

*Abbreviations*: *mRECIST* Modified Response Evaluation Criteria in Solid Tumors, *CR* Complete response, *PR* Partial response, *SD* Stable disease, *PD* Progressive disease

## Discussion 

Since BCLC-B HCC patients are heterogeneous in terms of tumor burden and hepatic reserve function, it remains challenging to make treatment decisions in these patients [7, 12]. We performed a head-to-head-comparison of the ALBI-TAE model and the other six scoring systems (Bolondi’s subclassification, HAP score, mHAP-II score, tumor burden score, SAT score, and SEC) in predicting HCC prognosis. We demonstrated that the ALBI-TAE model had the most predictive performance (Harrell’s C-index 0.633) in differentiating OS in BCLC-B HCC patients who underwent TACE. Moreover, the ALBI-TAE model was identified as an independent prognostic factor for survival outcome in multivariate analysis.

Compared with the other scoring systems based on tumor size and tumor number, the ALBI-TAE model, which consists of up-to-11 criteria, ALBI grade, and serum AFP level, offered a significantly higher C-index (0.633) than the SEC (0.578), SAT score (0.574), and tumor burden score (0.546). Our results confirmed the idea that tumor burden alone was insufficient to establish clear cut treatment decisions because HCC development was associated with liver cirrhosis in more than 80% of patients [4]. Most patients have two diseases that include HCC and liver cirrhosis and both impact an impaired prognosis. Currently, TACE is suggested for HCC patients with Child–Pugh class A and those with highly selected Child–Pugh class B cirrhosis [20]. As a result, a suitable selection of patients with BCLC-B HCC patients undergoing TACE remains challenging owing to different patient outcomes.

To address this issue, several scoring systems calculate the tumor burden and liver function, e.g., Bolondi’s subclassification, HAP score, and mHAP-II score [12, 16, 17]. However, our study illustrated that the ALBI-TAE model had the highest C-index among these scoring systems and represented better prognostication to differentiate survival outcome in BCLC-B HCC patients who underwent TACE. We demonstrated that the very high-risk patients in ALBI-TAE group D had a significantly higher risk of death than the high-, intermediate- and low-risk patients, which were ALBI-TAE groups C, B, and A, respectively. Therefore, the ALBI-TAE model can serve as a guide in the selection of optimal candidates for TACE.

Patients with high-risk (ALBI-TAE group C) and very high-risk (ALBI-TAE group D) hepatocellular carcinoma (HCC) have a median overall survival (OS) of 8–11 months, which is similar to patients with BCLC-C HCC receiving sorafenib treatment [21]. Furthermore, the response to TACE treatment is notably lower in ALBI-TAE group C (43%) and D (18%), respectively, and there is a high incidence of liver decompensation (9% and 29% in group C and D, respectively). Therefore, caution is advised when considering TACE as a treatment option for these patients. Instead, other alternative treatments such as radioembolization, targeted drugs, or immunotherapy should be considered rather than adhering to TACE until the disease progresses. These options may provide additional benefits and have the potential to improve outcomes for these patients. On the other hand, in the low-risk ALBI-TAE group A patients with an expected OS of 40.8 months, superselective TACE with achievement of portal vein visualization and circumferential safety margin is recommended to minimize local tumor recurrence and prolong survival outcome [22, 23]. However, if patients are eligible, other treatment modalities with a curative purpose, such as liver transplantation, resection, or ablation, should be done to reach a prognosis outcome comparable to BCLC-A patients. In addition, in the ALBI-TAE group B patients with an expected median OS of 20.1 months, TACE remains mandatory. These findings are consistent with a previous large systematic review that included 10,108 patients who underwent TACE [24].

The overall median OS results in our study were lower than the original Taiwan study (16.6 vs. 21.3 months) [18]. Also, by stratifying the ALBI-TAE model into groups A, B, C, and D, the median OS results in our study were lower than the original study with the exception of group D (our study: 40.8, 20.1, 10.6, and 7.5 months vs. original study: 65.9, 30.2, 17.4, and 6.0 months, respectively) [18]. The lower survival rates in our study were possibly due to the higher tumor burden and a lower proportion of Child–Pugh class A patients. The mean ± SD tumor size of the eligible patients in our study was 7.2 ± 4.6 cm compared with 6.7 ± 3.7 cm in the original Taiwan study. Notably, 60% of the patients in our study had large tumors > 5 cm. Moreover, a lower ratio of Child–Pugh class A patients were observed in our study in contrast to the original study (66% vs. 86%), which possibly led to the lower survival rates.

This study has a few noteworthy strengths. First, to the best of our knowledge, this was the first external validation study of the ALBI-TAE model in predicting the outcome of BCLC-B HCC patients undergoing TACE. Second, this study is a real-world evaluation in a heterogeneous population with various etiologies of HCC, i.e. hepatitis B, C, and alcohol, and relatively high tumor burdens. Third, our cohort compared seven scoring systems in patients who underwent conventional TACE in the entire cohort, which may be a potential benefit of this study.

Our study has a few notable limitations. First, this was a retrospective cohort study at a single medical center in Southeast Asia that should be validated in a prospective trial. Second, we did not analyze the expertise of the operators or the level of selective catheterization that might impact the survival outcome. Third, although the radiological response evaluation was recognized as an independent prognostic factor for survival in the multivariate analysis, it was not employed in the scoring system. The purpose of our study was to compare the ALBI-TAE model with six other scoring systems for efficient guidance in patient selection for TACE at the time of diagnosis before initiation of treatment.

## Conclusions

We demonstrated that the ALBI-TAE model displayed excellent differentiation in OS in patients who underwent TACE. Among the seven scoring systems, the ALBI-TAE model had the best predictive ability with the highest Harrell’s C-index in prognostication of survival outcome. The ALBI-TAE model is a simple and valuable prognostic tool to identify BCLC-B HCC patients who are suitable to receive TACE as the initial treatment.

## Data Availability

All data were stored separately in a data repository and are available from the corresponding author on reasonable request.

## Abbreviations

HCC: Hepatocellular carcinoma
BCLC: Barcelona Clinic Liver Cancer stage
TACE: Transcatheter arterial chemoembolization
OS: Overall survival
ALBI: Albumin-bilirubin grade
SAT: Six-and-twelve score
SEC: Seven-eleven criteria
HAP: Hepatoma Arterial-Embolization Prognostic score
mHAP: Modified Hepatoma Arterial-Embolization Prognostic score
AFP: Alpha-fetoprotein
mRECIST: Modified Response Evaluation Criteria in Solid Tumors
CR: Complete response
PR: Partial response
SD: Stable disease
PD: Progressive disease
ECOG: Eastern Cooperative Oncology Group
